# An Experimental and Finite Element Approach for a Better Understanding of Ti-6Al-4V Behavior When Machining under Cryogenic Environment

**DOI:** 10.3390/ma14112796

**Published:** 2021-05-24

**Authors:** Roland Bejjani, Charlie Salame, Mikael Olsson

**Affiliations:** 1 Department of Mechanical Engineering, Lebanese American University, Byblos P.O. Box 36, Lebanon; Charlie.salame@lau.edu.lb; 2 Materials Science, Dalarna University, SE-791 88 Falun, Sweden; mol@du.se

**Keywords:** titanium, CFD, FEM, cryogenic machining, cutting forces, tool wear

## Abstract

Due to increasing demand in manufacturing industries, process optimization has become a major area of focus for researchers. This research optimizes the cryogenic machining of aerospace titanium alloy Ti-6Al-4V for industrial applications by studying the effect of varying the nozzle position using two parameters: the nozzle’s separation distance from the tool–chip interface and its inclination angle with respect to the tool rake face. A finite element model (FEM) and computational fluid dynamics (CFD) model are used to simulate the cryogenic impingement of cryogenic carbon dioxide on the tool–workpiece geometry. Experiments are conducted to evaluate cutting forces, tool wear, and surface roughness of the workpiece, and the results are related to the CFD and FEM analyses. The nozzle location is shown to have a significant impact on the cutting temperatures and forces, reducing them by up to 45% and 46%, respectively, while the dominant parameter affecting the results is shown to be the separation distance. Cryogenic machining is shown to decrease adhesion-diffusion wear as well as macroscopic brittle chipping of the cutting insert compared to dry turning, while the workpiece surface roughness is found to decrease by 44% in the case of cryogenic machining.

## 1. Introduction

The modern manufacturing industry needs to meet the growing demands of aerospace production, while adhering to increasingly strict environmental and sustainability constraints, which calls for the optimization of the machining methods used in the industry. This improvement in productivity, which is defined as the production output in a given time frame, requires increasing the cutting speeds and feeds, which adversely affects the cutting temperatures and causes them to reach temperatures above 900 °C in the case of titanium alloy Ti-6Al-4V [1]. Titanium alloys are known for being difficult-to-machines alloys, specifically because of these high cutting temperatures which promote different types of tool wear and eventually lead to premature tool failure. The increased tool wear also compromises the surface integrity of the workpiece and the high cutting temperatures induce tensile residual stresses, as well as surface and subsurface cracks [2]. Therefore, cryogenic machining has become an area of vast investigation for researchers with potential machinability benefits.

Previous work showed important improvements when cryogenically machining titanium alloys compared to dry cutting. Experiments by Jerold and Kumar showed a decrease in cutting temperatures by up to 50% when using liquid carbon dioxide to cut Ti-6Al-4V compared to dry machining [3]. In cryogenic milling, Sadik and Isakson showed an increase in tool life by six times in cryogenic machining of Ti-6Al-4V compared to dry milling [4]. In addition, Bordin et al. showed a decrease in adhesion on the tool rake face when turning Ti-6Al-4V cryogenically compared to dry turning [5]. Cryogenic machining was also shown to have a positive effect on the surface roughness compared to flood machining, with a decrease of 19% in average surface roughness when using liquid nitrogen [6]. These papers demonstrate the cryogenic effect on the machining process; however, the optimization of the nozzle position is not accounted for, which is an objective of this research paper.

Few papers study the optimization of the nozzle position in a cryogenic system, but they are mostly simulation based. Previous work used a computational fluid dynamic (CFD) model to vary the nozzle position but simplified the tool–chip geometry into a flat Ti-6Al-4V plate and ignored the heat generated due to plastic deformation of machining [7]. Another research used a CFD model to simulate the correct tool–chip geometry but assumed a constant convection coefficient [6]. More recent work used a finite difference model to vary the cryogenic nozzle’s position between rake and flank face, but this model assumed only 1D heat transfer on a flat plate [8]. Thus, the literature lacks a model that accurately simulates the cryogenic environment and impingement of the cryogenic fluid on the tool–chip interface.

**Figure 1:**
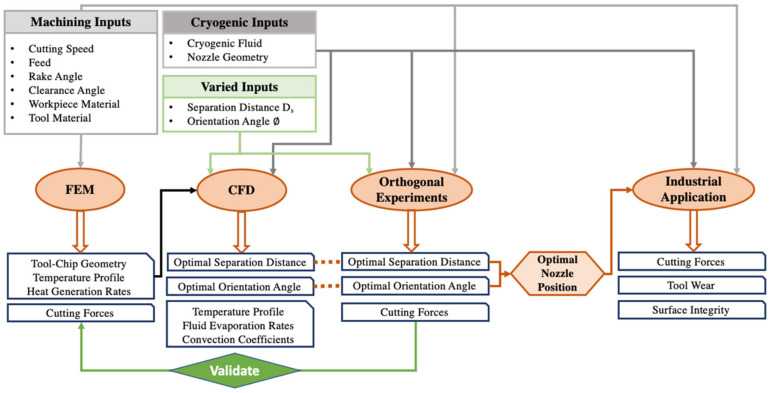
Methodology flowchart showing different parts of the research.

A cryogenic model that accurately simulates the impingement of the fluid on the tool–chip geometry is needed in order to better understand the effect of the nozzle location on the cryogenic machining process. To address this gap, this research uses a finite element model (FEM), CFD validated by experiments to determine the optimal nozzle position in the cryogenic setup, following the methodology illustrated in Figure 1 The term ‘optimal nozzle position’ in this research paper is used to refer to the optimal among the nozzle positions that were studied, and not the absolute optimal nozzle position. It should be noted that the optimal nozzle position for each cryogenic setup may be different, however, the results and interpretations made in this paper should remain valid.

**Figure 2:**
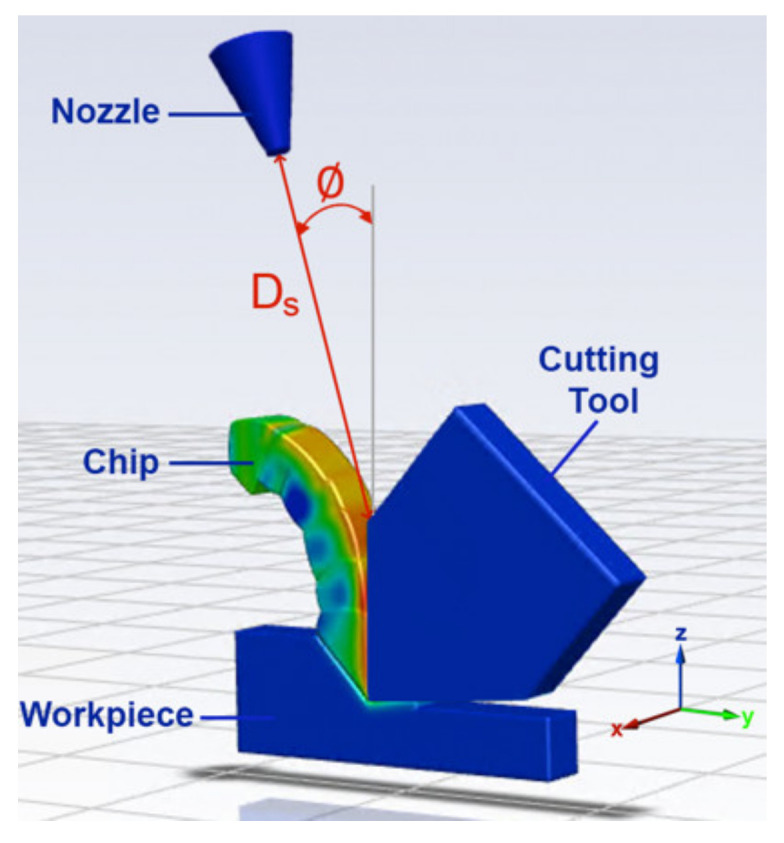
Tool–chip geometry showing the varied parameters, D_s_ and ∅.

The nozzle position in this research is optimized using two parameters, the separation distance D_s_ and the inclination angle ∅ from the rake face, shown in Figure 2. The developed FEM model simulates the orthogonal cutting of Ti-6Al-4V and outputs the cutting geometry, temperature profile, heat generation rate and cutting forces. These are then fed to the CFD model, as shown in Figure 1, where the cryogenic impingement on the tool–chip interface is simulated from different nozzle positions, varied in terms of D_s_ and ∅, to obtain the optimal temperature profile. By varying the same nozzle positions as the CFD model, the experimental section of the study aims to determine the nozzle position that yields the minimum cutting forces. As shown in Figure 1, the experimental results are linked to the CFD results and a correlation between the cooling rates and the cutting forces is established to determine the optimal nozzle position for cryogenic machining. This optimal nozzle position is then applied during longitudinal turning of Ti-6Al-4V, referred to as an industrial practical application in Figure 1, to determine the effect of using the optimized nozzle position on tool wear and surface integrity when applied on an industrial scale.

The research provides a better understanding of the cryogenic effect during machining of aerospace alloys, by linking the thermo-fluid properties of the cryogenic fluid to the heat transfer mechanisms at work during the cooling process. In addition, the work examines the effect of the cryogenic application on the tool wear mechanisms. Lastly, the research studies the effect of varying the nozzle position to provide an optimized industrial solution that reduces cutting forces, extends tool life and improves surface quality of the finished product.

## 2. Materials and Methods

### 2.1. FEM Model: Orthogonal Cutting of Ti-6Al-4V

A 2D FE model was developed on ABAQUS in order to simulate the orthogonal cutting of titanium alloy Ti-6Al-4V. The aim of the model is to obtain the heat generation rates and temperature profiles to be used in the CFD simulations, as well as the cutting forces to be validated using the experimental results.

#### 2.1.1. Geometry, Meshing and Setup

The FE simulations used a Lagrangian thermo-coupled dynamic explicit analysis with a quad-element CPE4RT mesh element. Simple Coulomb friction was used for the surface to node interaction, with a friction coefficient of 0.3, as recommended by Calamaz et al. [9]. The cutting speed was set as 90 m/min, the depth of cut as 0.1 mm and the feed as 0.1 mm/rev. The tool used had a 0° rake angle and a 5° clearance angle, matching the experiments conducted. The thermal conductivity, specific heat and density used for the workpiece were 7.3 W/mC, 570 J/kgK and 4512 kg/m^3,^ respectively, while those used for the cutting tool were 162 W/mC, 197 J/kgK and 15,800 kg/m^3^ [1].

#### 2.1.2. Models

The constitutive equation used to model material behavior in the FE model was the Johnson–Cook model equation shown in Equation (1), coupled with the Johnson–Cook damage model shown in Equation (2) [10],

(1) $$\bar{\sigma }=\left[A+B{\left(\bar{\varepsilon }\right)}^{n}\right]\left[1+C\ln\left(\frac{\alpha }{\beta }\right)\right]\left[1-{\left(\frac{T-T_{room}}{T_{melt}-T_{room}}\right)}^{m}\right]$$

where $\bar{\sigma }$ is the equivalent stress, *A* is the yield stress initially, *B* is the hardening modulus, *C* is the strain rate factor, *n* is the work hardening exponent, *m* is the thermal softening coefficient, *T_room_* is the room temperature and *T_melt_* the melting temperature.

(2) $$D=\sum \frac{\Delta \bar{\in }}{\left(D_{1}+D_{2}\exp D_{3}\frac{\sigma_{m}}{\bar{\sigma }}\right)\left(1+D_{4}\ln\frac{\alpha }{\beta }\right)\left[1-D_{5}\left(1-\frac{T-T_{room}}{T_{melt}-T_{room}}\right)\right]}$$

where $\Delta \bar{\in }$ is the increment in plastic strain, $\sigma_{m}$ is the average of the 3 normal stresses and $\bar{\sigma }$ is the von Mises equivalent stress, calculated from Equation (1). Fracture occurs and the element is removed from the computation when *D* reaches a value of 1.0 [11]. The Johnson–Cook material values *A*, *B*, *C*, *n* and *m* were set as 987.8 MPa, 761.5 MPa, 0.01516, 0.41433 and 1.516, respectively, while the damage model values *D*_1_, *D*_2_, *D*_3_, *D*_4_ and *D*_5_ were set as −0.09, 0.25, −0.5, 0.014 and 3.87, respectively [1].

### 2.2. CFD Model: Cryogenic Impingement of Ti-6Al-4V

After the orthogonal cutting of Ti-6Al-4V using the FEM model, the tool–chip geometry was modeled using ANSYS Fluent with a nozzle impinging cryogenic fluid onto the tool–chip interface from 4 different nozzle positions. The objective behind the CFD model was to obtain the velocity profiles of the flow, the temperature at the tool–chip interface, the evaporation rates of the cryogenic fluid and the heat transfer coefficient, all of which are extremely difficult to obtain experimentally. These results are required as they would allow more in-depth understanding of the mechanisms at work during cryogenic impingement in the cutting zone.

#### 2.2.1. Geometry and Setup

The 3D CFD model used for the simulations is shown in Figure 2. The geometry of the tool and chip were obtained from the cutting model and recreated for the CFD model to account for the geometry effect on the fluid flow. The temperature distribution profiles were obtained from the FEM results and surface fitted using a subroutine developed in MATLAB to yield distribution functions. For each section in the geometry, numerous nodes (*x_i_*, *y_i_*) were used and the equivalent temperature *T_i_* was obtained. Then, *x_i_*, *y_i_* and *T_i_* were input into 3 vectors and used to plot *T* in a polynomial surface fit of 3rd degree *x* and *y*. These functions were then used as the initial temperature profiles in all the CFD simulations, where the initial temperature in the z-direction was assumed to be constant. This process was repeated for all the sections of the cutting geometry.

The heat generation rates Q_R_ and Q_S_, obtained from the FEM study, were also included in the CFD simulation at the primary and secondary shear zones and assumed to be constant throughout the simulation time. Heat transfer by conduction is activated and heat transfer is allowed between the tool, chip and workpiece using coupled walls in the simulation.

The other mode of heat transfer present in the simulation is convective heat transfer due to the jet of cryogenic CO_2_. The nozzle inlet pressure of the fluid is set to be 8 bars and assumed to be purely liquid (volume fraction = 1) upon entry to the nozzle. The fluid then undergoes evaporation as it makes its way from the nozzle to the tool–chip geometry. The liquid starts evaporating inside the nozzle as it collects heat from the surrounding environment, and then undergoes large rates of evaporation with the expansion of the fluid as it leaves the nozzle. The atmosphere between the nozzle and the tool–chip geometry is also a medium for the liquid CO_2_ to evaporate, until the remaining cryogenic CO_2_ meets the high temperatures near the tool–chip interface and exhibits high rates of evaporation.

In order to select which parameters to vary in this research, a preliminary CFD study was conducted on ANSYS where several nozzle parameters were tested and the ones that showed the biggest effect on the temperature distribution were chosen to be investigated in this research. In that study, the varied parameters were D_s_, ∅ and the angle β, measured from the yz-plane (shown in Figure 2), where the variation in β was determined to have less effect on the cutting temperatures. Therefore, due to limitations in computational resources and time, the studied parameters were limited to D_s_ and ∅.

Previous work with pressurized nozzle jet on a plate with varying distance showed that decreasing D_s_ beyond a certain minimum will decrease the cooling effect of the cryogenic application due to the non-linear nature of the cryogenic cooling effect [12]. To determine the smallest possible Ds, we considered a physical limitation in our setup, where the closest distance at which the nozzle can be placed without interfering with the tool–workpiece geometry and the flow of chips was found to be 2 cm. The other extreme value was chosen to be 4 cm, as beyond that separation distance, the nozzle no longer had a focused impingement on the tool–chip interface, thus decreasing its cooling effect. A similar logic was applied to determine the two extreme values for the orientation angle (the parameters are shown in Table 1).

#### 2.2.2. Governing Equations in Fluid Dynamics

The CFD simulations conducted on ANSYS Fluent were governed by the continuity, momentum and energy equations that constitute the main equations that govern fluid dynamics. The turbulence model selected was the realizable k-epsilon model, which is widely used in simulating industrial flows [10]. The multiphase model chosen was the volume of fluid model, which allows accurate computation of the phase change in the model.

##### Turbulence Model: Realizable k-Epsilon

The realizable k-epsilon computes 2 equations: the equation for turbulence kinetic energy *k* and that for dissipation of turbulence energy ε, shown in Equations (3) and (4) respectively [10],

(3) $$\frac{\partial }{\partial t}\left(\rho k\right)+\frac{\partial }{\partial x_{j}}\left(\rho ku_{j}\right)=\frac{\partial }{\partial x_{j}}\left[\left(\mu +\frac{\mu_{t}}{\sigma_{k}}\right)\frac{\partial k}{\partial x_{j}}\right]+G_{k}+G_{b}-\rho \varepsilon -Y_{M}+S_{k}$$

where ρ is the density, μ is the molecular viscosity, *u_j_* is the velocity projection in the corresponding direction, and σ*_k_* is the turbulent Prandtl number. $G_{k}$ and $G_{b}$ are turbulence kinetic energies due to the mean velocity gradients and buoyancy, and $S_{k}$ is a source term. *Y_M_* represents the fluctuating dilatation in compressible turbulence to the overall dissipation rate.

(4) $$\frac{\partial }{\partial t}\left(\rho \varepsilon \right)+\frac{\partial }{\partial x_{j}}\left(\rho \varepsilon u_{j}\right)=\frac{\partial }{\partial x_{j}}\left[\left(\mu +\frac{\mu_{t}}{\sigma_{\varepsilon }}\right)\frac{\partial \varepsilon }{\partial x_{j}}\right]+\rho C_{1}S_{\varepsilon }-\rho C_{2}\frac{\varepsilon^{2}}{k+\sqrt{v\varepsilon }}+C_{1\varepsilon }\frac{\varepsilon }{k}C_{3\varepsilon }G_{b}+S_{\varepsilon }$$

where $C_{1}=\max\left[0.43,\frac{\eta }{\eta +5}\right]$, $\eta =S\frac{k}{\varepsilon }$, $S=S_{ij}\sqrt{2}$, *S_ij_* is the mean strain rate, η is the effectiveness factor, ν is the kinematic viscosity, $C_{2}$ and $C_{1\varepsilon }$ are constants, $\sigma_{\varepsilon }$ and σ*_k_* are the turbulent Prandtl numbers, and $S_{\varepsilon }$ is a source term.

##### Multiphase Model: Volume of Fluid (VoF)

The VoF model solves a volume fraction continuity equation for each secondary phase in the system, shown in Equation (5) [10]. For the primary phase, ANSYS calculates the Volume Fraction based on the condition that the sum of all phases should amount to 1. The equations are solved by an explicit time discretization with implicit body forces.

(5) $$\frac{1}{\rho_{q}}\left[\frac{\partial }{\partial t}\left(\alpha_{q}\rho_{q}\right)+\nabla \cdot \left(\alpha_{q}\rho_{q}\vec{v_{q}}\right)=S_{\alpha q}+\sum_{p=1}^{n}\left({\dot{m}}_{pq}-{\dot{m}}_{qp}\right)\right]$$

where $\alpha_{q}$ is the phase-*q* volume fraction, $\rho_{q}$ is the phase’s density, ${\dot{m}}_{pq}$ is the mass transferred from phase *p* to phase *q* and ${\dot{m}}_{qp}$ from phase *q* to *p*. The mass source term $S_{\alpha q}$ is zero.

### 2.3. Experiments

In addition to the cryogenic simulations, turning experiments that varied the same nozzle positions as the simulations were conducted. First, orthogonal turning was used to compare the cutting forces between the 4 different nozzle positions and the dry turning. Then, the tool wear in the cutting inserts for the optimal cryogenic case and dry turning was studied. Once the optimal nozzle position was determined, longitudinal turning was used to compare dry to cryogenic turning using the optimized nozzle location. The objective of these longitudinal experiments was to compare tool wear and surface roughness of the workpiece between dry and cryogenic turning in an industrial-like application.

#### 2.3.1. Design of Experiments

Table 2 summarizes the list of experiments conducted. The cutting parameters in the FE model match the experiment parameters for validation purposes. The cutting speed and feed were the same for all the experiments conducted: 90 m/min and 0.1 mm/rev, respectively, and the rake and clearance angles were set as 0° and 5°. The depth of cut for the orthogonal and longitudinal turning experiments was 0.1 mm. The nozzle position is varied according to the parameters shown in Table 2, which are the same nozzle positions simulated with the CFD model.

#### 2.3.2. Experimental Setup

**Figure 3:**
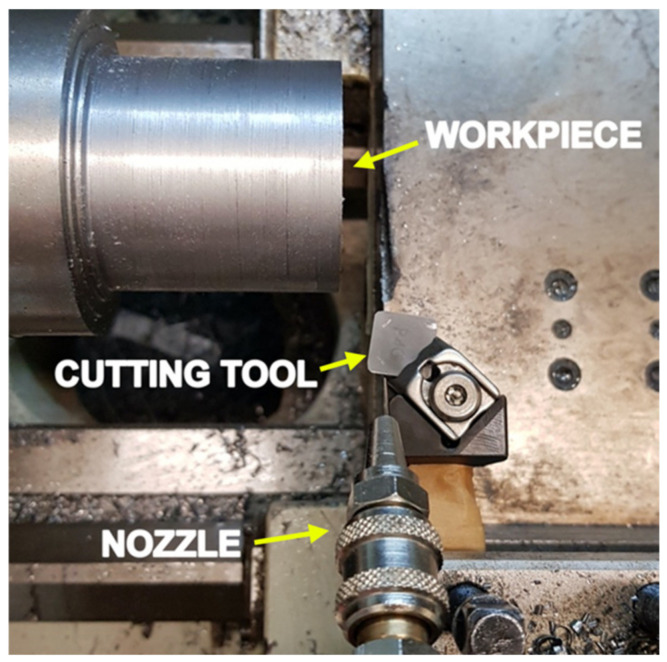
Experimental setup.

The experimental setup is shown in Figure 3. The Siemens Sinumerik 808D lathe was used to perform the machining, with a PCLNR/L 2020k12 modified tool holder using uncoated tungsten carbide tools (Sandvik Coromant CNMG 120,404 H13A). The tool holder was modified and mounted with strain gauges to match the smart tool holder designed by Zhao et al. [13], capable of accurately measuring the axial and tangential cutting forces after successful calibration. Each turning test used a brand-new cutting edge to guarantee matching initial conditions for all experiments. The cryogenic fluid was delivered to the tool–chip interface using an external cryogenic supply system, consisting of a converging nozzle, insulated pipe, pressure regulator and pressurized tank. The system supplies the fluid at a pressure of 8 bars through the 1-mm exit diameter of the converging nozzle. The surface roughness of the finished workpiece was measured with a Mitutoyo Profilometer SJ-210, while the scanning electron microscope images and the EDS analysis were obtained using the COXEM SEM machine.

## 3. Cutting Simulation and Cryogenic Impingement Model

### 3.1. Cutting Simulation FEM Results

**Figure 4:**
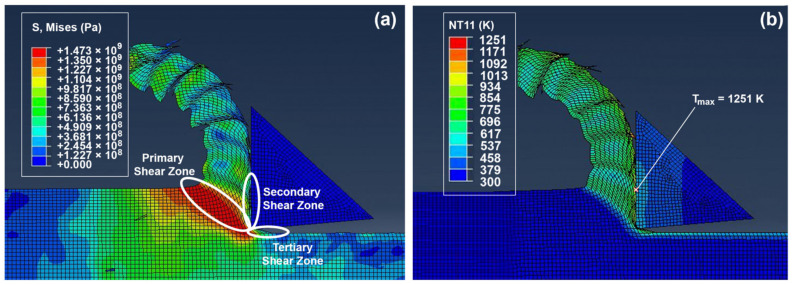
(**a**) Von Mises stress plot (in Pa) and (**b**) temperature profile (in K).

Figure 4a shows the Von Mises stress plot, while Figure 4b shows the temperature profile obtained during the orthogonal cutting of Ti-6Al-4V. The chips resulting from the cut are segmented, which is characteristic of machining titanium alloys due to the non-uniform deformation and shear localization at high cutting temperatures. Figure 4a plots the stress distribution in the system showing the highest stress at the primary shear zone (PSZ), where the most deformation occurs, followed by the secondary shear zone (SSZ) and lastly the tertiary shear zone (TSZ). From the FEM study, the tangential cutting force is *F_v_* = 471 N and the axial cutting force is *F_s_* = 286 N.

The temperature profile obtained during the cutting process was plotted in Figure 4b and shows the maximum temperature on the tool–chip interface with a value of 1251 K. The peak in temperature at the tool–chip interface is due to the heat generated at the secondary shear zone, the heat carried from the primary shear zone by the flowing chip, and the frictional contact between the chip and the tool.

Given the cutting parameters and the obtained cutting forces, it becomes possible to calculate the heat generation rates at the primary and secondary shear zones. The heat generation in the tertiary shear zone is minimal [14], and can be ignored. Equation (6) calculates the heat generated in the PSZ as [14],

(6) $$Q_{R}=\frac{F_{v}V}{60}$$

where $Q_{R}$ is the heat generated in the PSZ, *V* is the cutting velocity in m/min, and $F_{v}$ is the tangential cutting force in N. The heat generated in the SSZ is given by Equation (7) [14],

(7) $$Q_{S}=\frac{V\left(F_{v}\sin\alpha +F_{s}\cos\alpha \right)}{60r}$$

where *Q_S_* is the heat generated in the SSZ, *r* is the chip thickness ratio, α is the rake angle and *F_s_* is the axial force. The calculations yield *Q_R_* = 0.706 kW and *Q_S_* = 0.476 kW.

### 3.2. Cryogenic Impingement CFD Results

**Figure 5:**
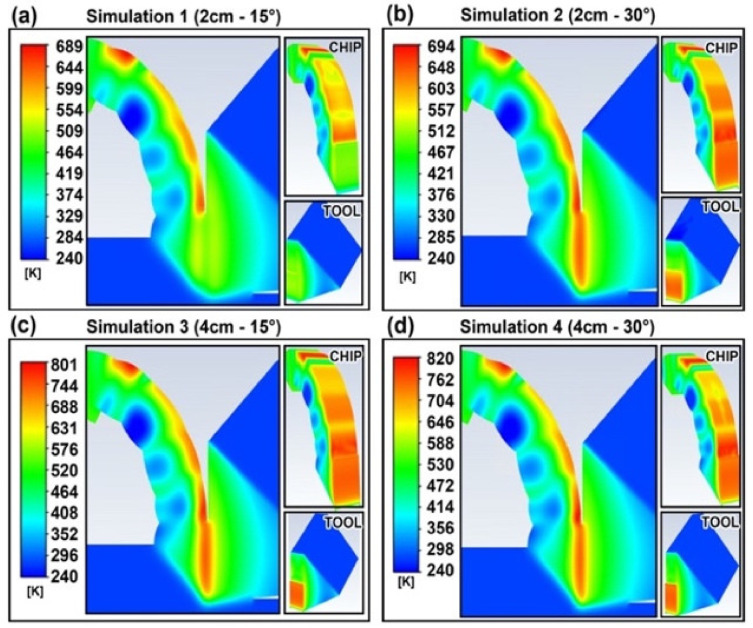
Temperature Profiles (in K).

**Figure 6:**
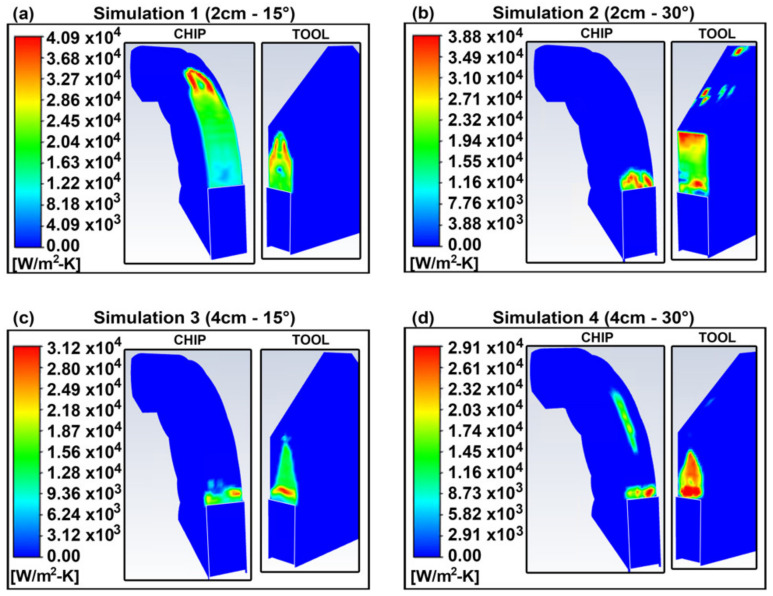
Heat convection coefficient profiles (in W/m^2^-K).

The temperature profiles after the impingement of the cryogenic jet are shown in Figure 5. There is a significant reduction in the temperature, where simulations 1, 2, 3 and 4 led to a 45%, 44%, 35% and 34% drop in the maximum temperature, respectively. The observed drops in temperature can be attributed to the high convective heat transfer that the impingement of the cryogenic fluid has on the cutting area, as shown in Figure 6.

**Figure 7:**
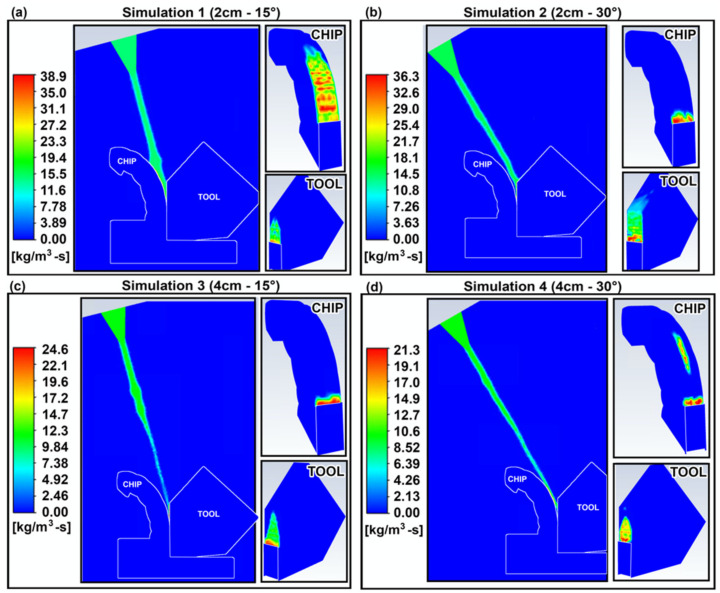
Evaporation rates (in kg/m^3^-s).

The liquid and gas stream of CO_2_ that come in contact with the higher temperature workpiece absorb heat from it, thus cooling it down. In particular, the high heat transfer from the tool–chip zone to the liquid CO_2_ particles, cause the cryogenic liquid to evaporate, enhancing the cooling effect by absorbing the latent heat of vaporization required to undergo this phase change. The rates of this evaporation are shown in Figure 7, where the evaporation rates in simulations 1 and 2 are higher than those in 3 and 4.

**Figure 8:**
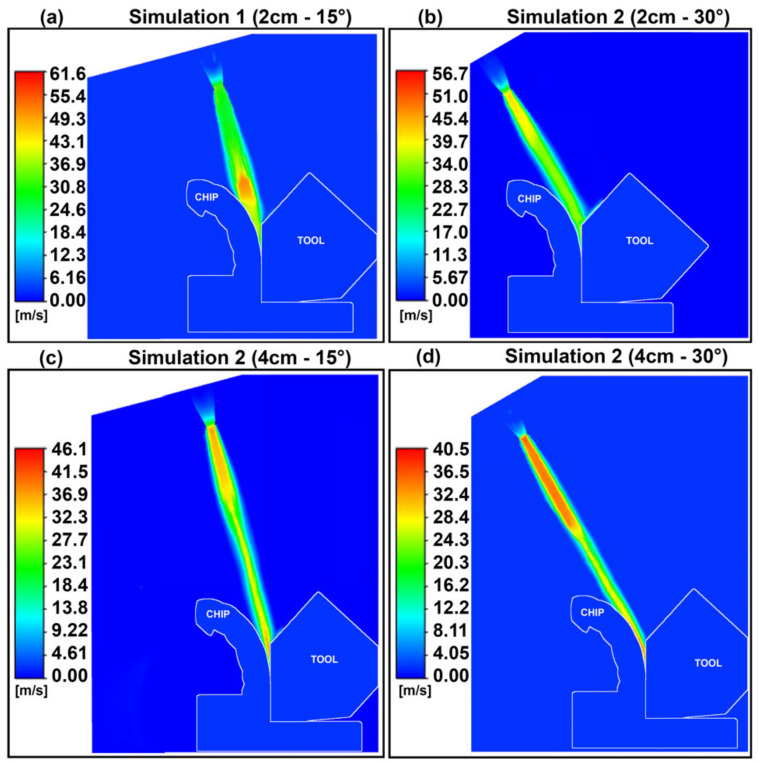
Jet velocity profiles (in m/s).

The optimal temperature profile was obtained in simulation 1, indicating that the shorter separation distance and less inclined nozzle led to a better cooling effect. This can be explained by the higher velocity near the tool–chip interface obtained in simulation 1, shown in Figure 8. The higher velocity of the fluid generates more turbulence, as indicated by the flow’s Reynolds number (Re) which increases with increasing velocity. The higher turbulence causes more mixing of the fluid and higher heat transfer via transport eddies and thus leads to a higher convection coefficient. This is supported by the convection coefficient plots of Figure 7, of which trends match those of the velocity profiles.

**Figure 9:**
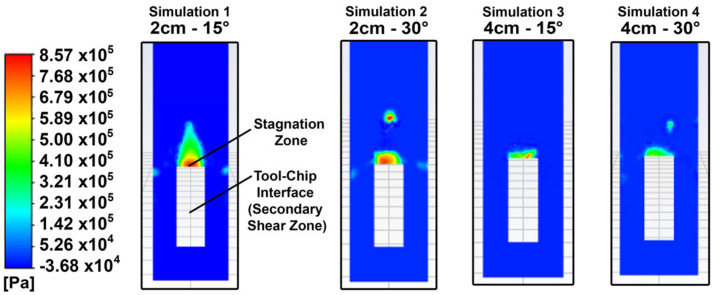
Static pressure at the cross section of the tool–chip interface (in Pa).

The fluid in the stagnation zones of simulations 1 and 2 has a higher localized static pressure, as shown in the plots of Figure 9, which shows the static pressure at the cross-section at the tool–chip interface. Simplifying the flow to an incompressible and isothermal flow, the Bernoulli equation can explain the fact that a decrease in flow velocity from a point to another downstream leads to an increase in its static pressure [15]. Thus, the areas which exhibit the highest static pressure are the areas where the fluid has its lowest velocity—the stagnating fluid. The stagnation zone of an impinging jet is the area that exhibits the maximum heat transfer due to the very thin boundary layer formed around its stagnation point [16]. Shademan et al. showed that during the impingement of a fluid on a flat plate, a lower value of D_s_/D, where D is the diameter of the nozzle (constant in this case), leads to a larger distribution of the stagnation zone with a higher pressure than lower D_s_/D cases [17].

At the higher static pressures in for nozzle positions 1 and 2, the saturation temperature required for phase change increases as well [18]. Thus, the particles of the cryogenic liquid need to absorb more heat to evaporate and would last longer in the liquid phase, which has considerably higher thermal conductivity and dynamic viscosity compared to gas CO_2_, therefore enhancing the cooling effect. This is further supported by the highest rates of evaporation observed in simulations 1 and 2 observed in Figure 7. Decreasing the angle ∅ for the same D_s_ had a minimal effect on the drop in temperature, but still led to a further decrease in the maximum temperature as seen in Figure 5. In the case of the higher angle ∅, the decreased cooling effect can be attributed to the fluid sliding off the geometry more easily which would decrease the static pressure and static zone, thus decreasing the areas of maximum heat transfer and consequently the cooling effect.

## 4. Experimental Results and Discussion

The cutting forces were measured during the orthogonal and longitudinal experiments and compared to the dry turning. The measurements during the orthogonal cutting were used to determine the nozzle position that led to the minimum cutting forces. The tool wear was also assessed for the optimal cryogenic case and compared to dry turning. Lastly, the surface roughness parameter *R_a_* was evaluated for the workpiece in longitudinal cryogenic and dry turning.

### 4.1. Cutting Forces

**Figure 10:**
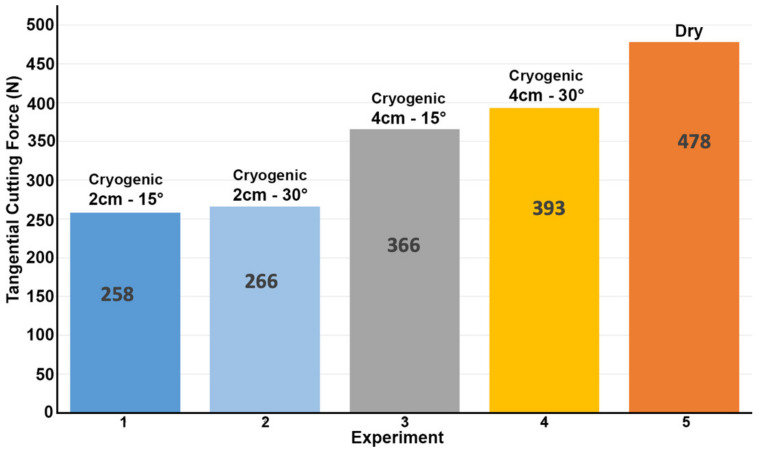
Tangential cutting forces during orthogonal turning of Ti-6Al-4V.

Figure 10 shows the measured tangential cutting forces for each experiment. Comparing the dry cutting measured tangential force and the FEM tangential force, the FEM forces are 2% less than the experimental one, which validates the FEM model.

The largest decrease in cutting forces compared to dry turning was measured for experiment 1, where the tangential cutting force was reduced from 478N by 46% to 258N. In the cases of experiments 2, 3 and 4, the percentage reductions in force were 44%, 23% and 18%, respectively. Therefore, the optimal nozzle positions in the experiments and the CFD simulations are in agreement. The nozzle position that resulted in maximum cooling (2 cm/15°) in the CFD simulation is the same position that showed the minimum cutting force during orthogonal cutting. In addition, as the cutting temperature decreased, the measured cutting force decreased as well, where T_Sim1_ < T_Sim2_ < T_Sim3_ < T_Sim4_ and F_Exp1_ < F_Exp2_ < F_Exp3_ < F_Exp4_. It is therefore possible to deduce that the cooling effect is in direct correlation with the cutting force.

Compared to dry turning, the cutting forces were reduced for all the experiments with cryogenic application. The drop in cutting forces can be attributed to the cooling effect that the cryogenic fluid has on the cutting insert, which decreases the thermal-induced softening of the insert and helps retain a sharp cutting edge for a longer period of time. Tool wear analysis was conducted in Section 4.2 and showed that cryogenic application does indeed preserve a sharp cutting edge for a longer time. This cooling effect is also enhanced by the significant difference in thermal conductivities between the workpiece and insert, indicating that the cryogenic fluid can absorb heat faster from the insert than the workpiece, thus cooling the insert at a faster rate and decreasing the thermal softening effect further.

**Figure 11:**
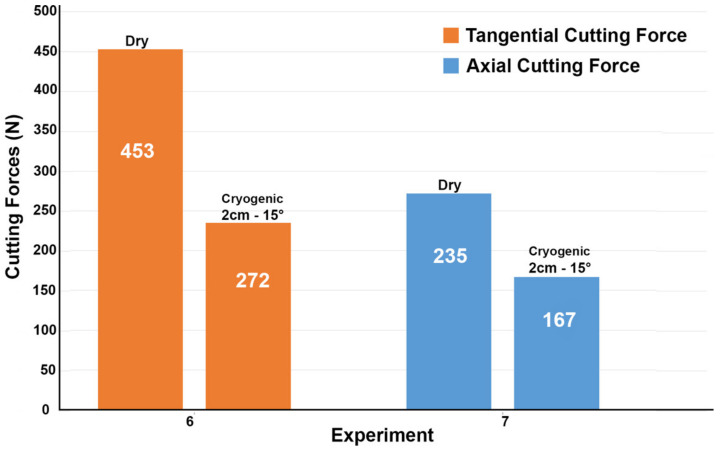
Tangential and axial cutting forces during longitudinal turning of Ti-6Al-4V.

The optimal cryogenic nozzle position was then applied for longitudinal turning and the axial and longitudinal forces were measured and compared to dry longitudinal turning. The results are shown in Figure 11, where the axial cutting force underwent a 38% drop to 167N and the tangential cutting force a 47% drop to 235N, a similar percentage drop compared to the orthogonal cutting case, confirming the benefits in force reduction found in orthogonal cutting.

### 4.2. Tool Wear

A qualitative study of the tool wear was completed for the case of dry turning and the optimal cryogenic case, in both the orthogonal cutting (experiments 1 and 5) and longitudinal turning (experiments 6 and 7). The wear was evaluated using optical microscopy, SEM and EDS analysis.

#### 4.2.1. Tool Wear during Orthogonal Turning

When machining titanium alloys with uncoated tungsten carbide tools, adhesion and diffusion are the most dominant wear mechanisms that lead to tool failure [19]. These wear mechanisms are influenced by the mechanical properties of titanium, which possesses low thermal conductivity, high hardness and high affinity to other elements at increased temperatures. Adhesion of workpiece material onto the tool is a common phenomenon observed during the machining of titanium alloys. Machining titanium produces thin chips, high cutting pressures at the tool–chip interface as well as high cutting temperatures. The low thermal conductivity of Ti-6Al-4V allows for high cutting temperatures, also accelerating the diffusion–dissolution phenomenon which is a thermally activated process and commonly observed during machining of titanium alloys [5].

**Figure 12:**
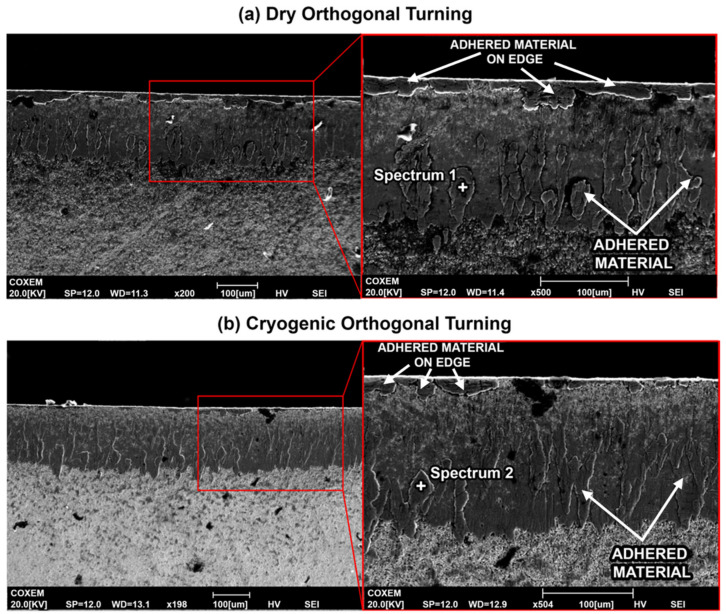
SEM images of the rake face of the (**a**) dry insert and (**b**) optimal cryogenic insert.

Figure 12 compares the inserts used in machining under dry and cryogenic conditions. It can be observed that more adhered workpiece material is present on the edge of the insert. Previous work when machining a similar workpiece indicated that small microchipping occurs on the edge very early on when machining Ti-6Al-4V [20,21]. The rough surface produced after the small chip provides additional anchoring for the workpiece material and promotes adhesion. This explains why more adhered material is present for the dry insert (Figure 12a), whereas under cryogenic conditions, the edge is preserved for a longer time with reduced microchipping on the edge.

The adhesion of the workpiece material to the tool substrate promotes chemical transfer between the two materials in a process called diffusion. The void created by microchipping, under the effect of high cutting pressure from the flowing chips promotes adherence of workpiece material which are then plastically deformed onto the tool and appear as smearing, as observed in Figure 12. The process can be repeated for different locations, and results in more signs of adherence on the insert due to the effect of flowing chips [22]. Adhesion and diffusion work hand in hand to accelerate the wear, where diffusion can deplete the carbon from the tool matrix, making it easier for the flowing chips to pull out the tungsten particles that are no longer anchored [23,24]. Figure 12 shows adhesion on the dry cutting insert, where the workpiece material is bonded to the tool rake face. Since diffusion is a wear mechanism that highly depends on the temperature at the cutting zone, the high cutting temperatures in the case of dry turning titanium provide ideal conditions for the exchange of elements between the tool and workpiece.

EDS spectra were taken at the same distance from the cutting edge and the results are summarized in Table 3, where spectrum 1 shows high concentrations of titanium, aluminum and vanadium on the cutting insert used for dry cutting. This shows the high level of adhesion that is a common characteristic of titanium machining. The insert used for cryogenic turning also shows the workpiece elements, but to a lesser extent than the dry insert. Concentrations of workpiece elements (Ti, Al and V) at spectrum 2 are considerably less, showing higher concentrations of tool elements W and Co instead. The decrease in Ti, Al and V concentrations using the EDS spectra is an indication of reduced adhesion when cryogenic fluid is applied.

**Figure 13:**
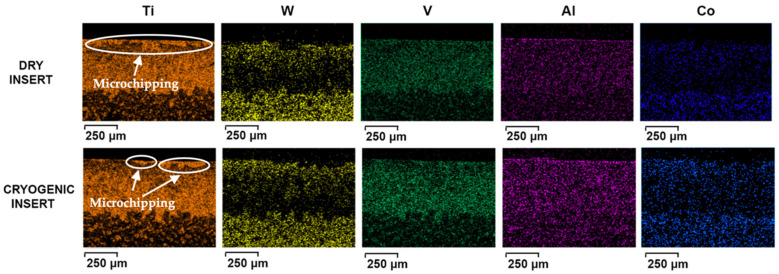
EDS element maps for dry and cryogenic inserts.

The EDS element maps in Figure 13 show the high concentration of titanium present on the rake face of the tool, confirming the high amount of adhesion during the machining process. While this benefits the tool by forming a layer to protect the substrate from abrasive wear, it actually promotes wear on the crater side by activating the diffusion mechanism as described previously. Instead of being plastically deformed onto the rake face, an alternative scenario is for the built-up Ti-6Al-4V lumps to break off regularly due to the high pressure and cutting forces exerted on the tool from the rotating workpiece, causing microchipping of the cutting edge. The microchipping can be observed on the cutting edges in both the dry and cryogenic inserts in Figure 13. Since adhesive wear is decreased in the case of cryogenic machining, then the frequency of microchipping is also reduced, as shown in Figure 13. This may explain why chipping can be observed along the entire cutting edge in the case of dry cutting but less so in the case of cryogenic turning. The size of the chips along the edge is also larger in the case of the dry cutting and this could be justified by the higher cutting forces in dry cutting as demonstrated previously, which are likely promoting the pulling out of the adhered particles, thus creating larger chipped zones [21]. This suggests that adhesion, combined with the higher cutting forces, can be the driving force behind the microchipping seen in Figure 13.

**Figure 14:**
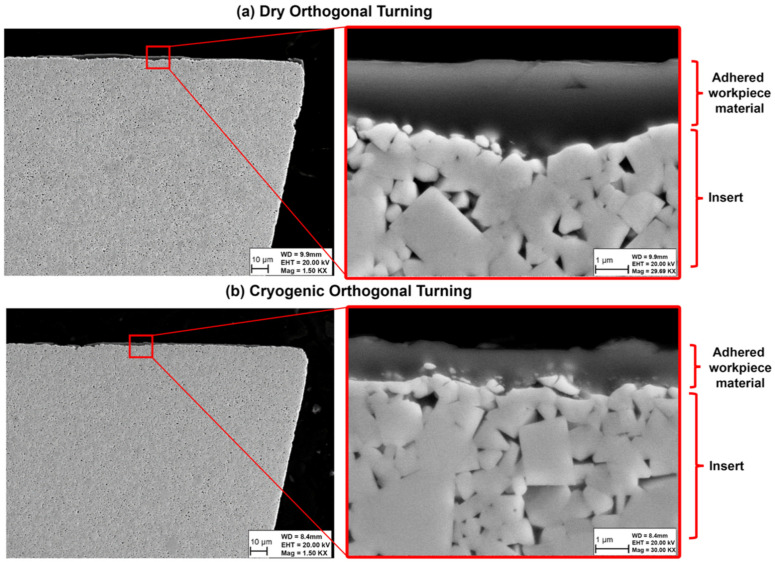
SEM images for the cross-sections of the (**a**) dry and (**b**) cryogenic cutting inserts.

From the SEM images for the cross sections of the cutting inserts, shown in Figure 14, it is evident that the depth of wear and adhered workpiece material on the rake side of the tool for dry cutting is larger than that for cryogenic turning. Under dry conditions, the worn rake face surface has deeper pits. In both cutting conditions, WC fragments can be observed. Since the cryogenic insert is still in the early stages of the wear process, more fragments can be observed compared to the dry cutting condition.

#### 4.2.2. Tool Wear during Longitudinal Turning

**Figure 15:**
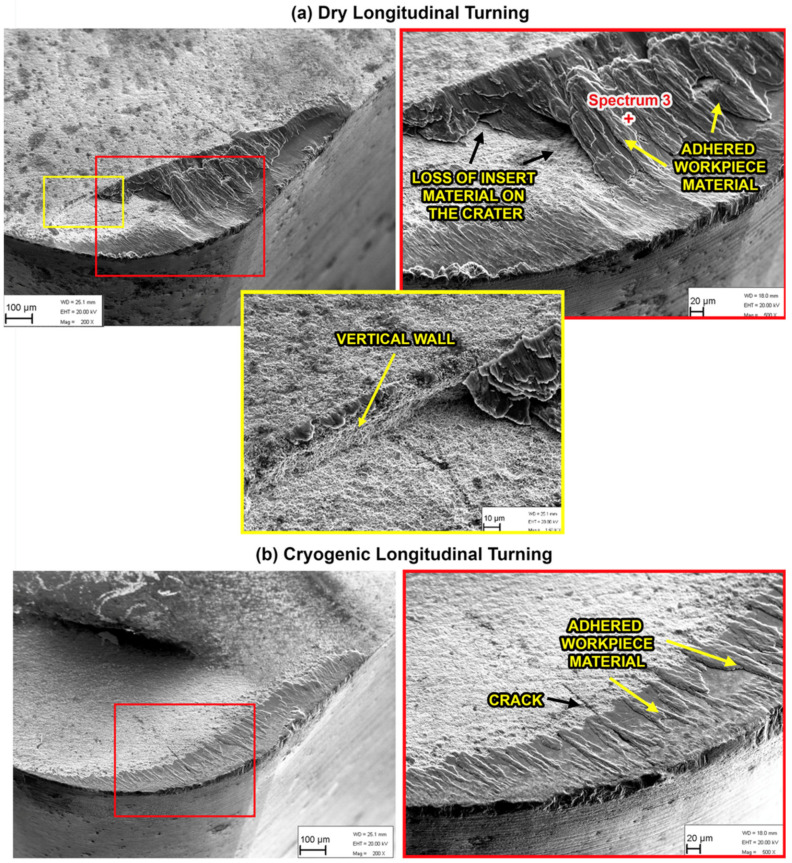
SEM images of the insert rake face for (**a**) dry and (**b**) cryogenic longitudinal turning.

The experiments of longitudinal turning were used to compare the tool wear in dry turning versus the optimal cryogenic case. The tool wear observed using SEM for the cutting inserts used in the longitudinal experiments is shown in Figure 15. Similar to the case of orthogonal cutting, there is significant adhesion observed, in addition to loss of insert material on the crater face of the tool. The EDS elemental maps show the high levels of adherence of the Ti-6Al-4V workpiece to the tool, while Table 4 presents the elemental concentrations obtained at spectrum 3, showing the existence of workpiece material and high adhesion under dry cutting conditions.

**Figure 16:**
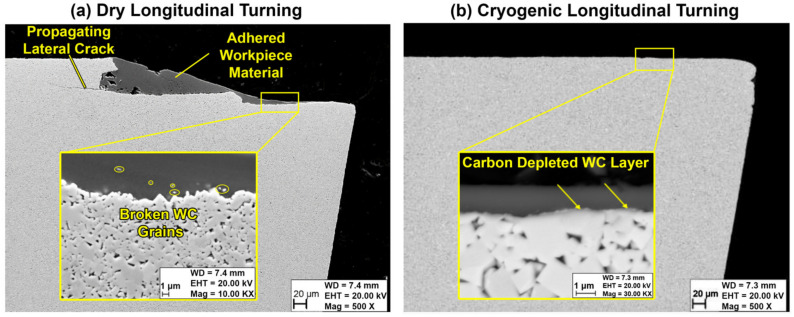
SEM Images for the cross-sections of the (**a**) dry and (**b**) cryogenic cutting insert.

From the SEM images in Figure 15, it is evident that the dry turning led to significant volume loss on the crater side compared to reduced loss in the case of cryogenic turning for the same machining parameters and duration. The loss of insert material can be related to brittle-like macro-chipping resulting in the formation of a vertical wall in the crater face surface, as shown in Figure 15a for the dry cutting insert. In the case of cryogenic turning, the insert also shows signs of cracking; however, the cracks are normal to the surface and to the cutting edge and are less dangerous than the lateral cracks. The lateral cracks can be observed in Figure 16, which shows the cross section of the cutting inserts for both dry and cryogenic machining. The reason for the observed lateral cracks and loss of cutting edge in the case of dry turning is possibly due to the micro-chipping and adhesion–diffusion mechanisms at work. The higher thermal load in the dry cutting is causing a higher thermal gradient in the insert. The resulting expansion of the insert material may then lead to higher stresses, which can lead to the dangerous lateral cracks that gradually cause the loss of the cutting edge.

The loss of material on the crater face may be aggravated by the synergy between diffusion and adhesion wear mechanisms. According to Saketi et al., when the Ti-6Al-4V adheres to the tool rake face, the tool substrate elements diffuse to the adhered layer, especially carbon, causing the embrittlement of the tool matrix [23]. The carbon depletion and decrease in binder concentration in the insert makes it easier for the flowing chips to pull out the adhered Ti-6Al-4V layer and with it, pull out weakened tungsten carbide grains leading to considerable material loss in the cutting tool. The carbon-depleted layer can be seen in the SEM image for the rake face of the cryogenic insert in Figure 16b. Other previous work showed a depleted carbon layer on the surface with high W content and was shown as a lighter shade in the SEM images [25]. Figure 16b shows a similar pattern. A model by Saketi et al. describes the removal of the brittle depleted region where scattered pieces from the depleted surface are gradually removed by the flow of chips [24]. Figure 16a shows similar scattered pieces of WC which are observed inside the workpiece material. Comparing Figure 16a,b indicates that the cryogenic cooling reduces the micro-removal from the tool surface.

**Figure 17:**
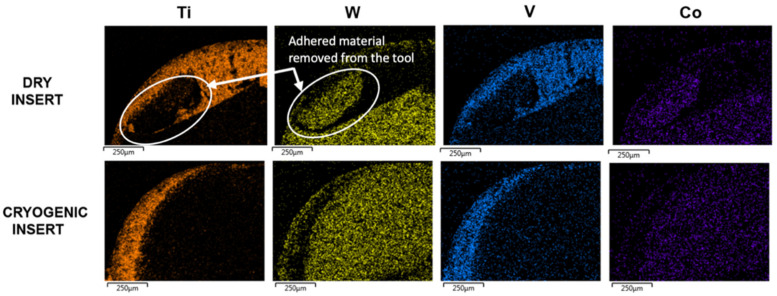
EDS element maps for dry and cryogenic inserts.

**Figure 18:**
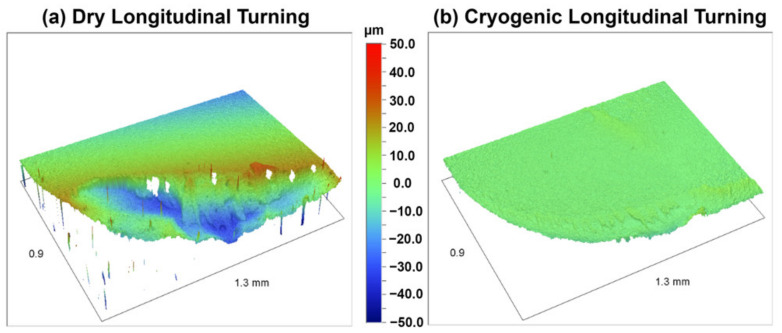
Topography worn volume difference between (**a**) dry and (**b**) cryogenic longitudinal turning.

The EDS mapping for the dry cutting insert, shown in Figure 17, shows the adhesion mechanism of Ti-6Al-4V elements on the crater. The substrate material is exposed in the crater, which indicates that the adhered layer was pulled off exposing the tungsten. This wear on the crater face is slowed down/eliminated in the case of cryogenic machining due to the reduction in temperature which considerably slows down both adhesion and diffusion, effectively reducing the main causes that trigger these wear mechanisms. The decrease in worn volume on the crater is shown by difference in the 3D topographic worn volume between the two inserts, as shown in Figure 18.

**Figure 19:**
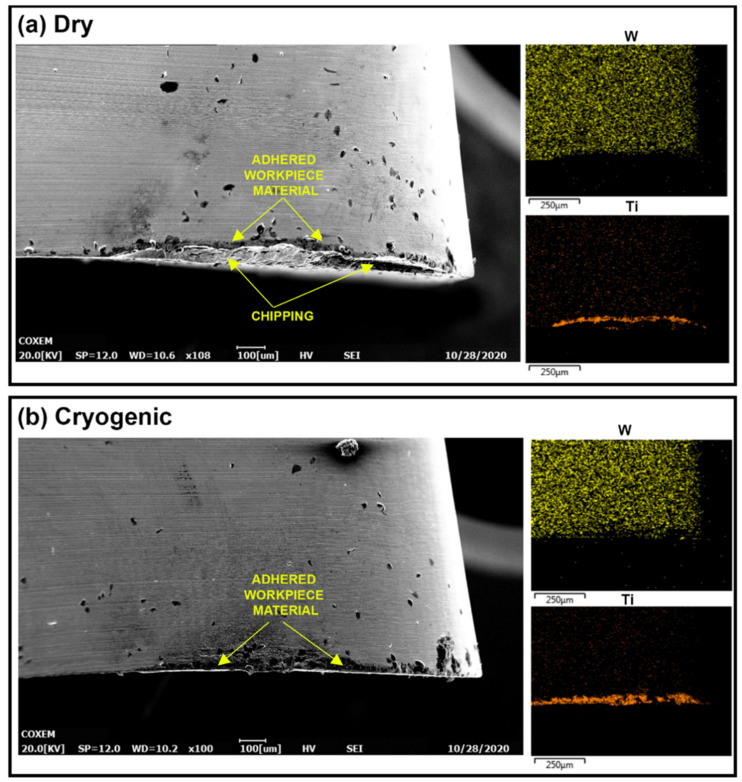
SEM and EDS element maps for the flank face in (**a**) dry and (**b**) cryogenic longitudinal turning.

On the flank side of the inserts, the dominant type of wear is typically abrasive wear due to the flowing chips and high friction between the workpiece and cutting insert [4]. During the machining of titanium alloys, adhesion and diffusion act simultaneously in addition to abrasion wear and increase flank wear considerably. The adhesion of titanium on the insert is observed at the flank face of both inserts in the EDS element maps of Figure 19. The SEM images in Figure 19 also show that most of the edge is chipped in the case of dry turning, which is not apparent in the case of cryogenic turning. The chipping in the case of dry turning can be caused by the combination of high stresses and adhesion as described previously [26]. The EDS maps in the case of cryogenic turning show that the bottom of the tungsten distribution is nearly a straight line, whereas the maps for dry turning show an oblique edge, indicating that the cutting edge has been lost in the case of dry turning. The oblique contour is what is left from the edge—below this line, the EDS will not detect signals arriving from a different plane. This may also explain why the EDS maps show a higher concentration of titanium in the case of cryogenic machining. The titanium elements in the dry insert are adhering to the broken cutting edge which is now in a different plane and are no longer being captured by the EDS imaging.

### 4.3. Surface Roughness of Workpieces

In industrial applications, one of the most important outcomes of a machining process is the quality of the finished workpiece. For this reason, surface integrity is evaluated and includes surface roughness, residual stresses and other parameters. In this research, the focus was on evaluating the average surface roughness parameter *Ra* of the turned titanium workpiece in both dry and cryogenic environments in order to determine the effect of cryogenic application on the average surface roughness parameter *Ra*.

The average surface roughness *Ra* for the cryogenic workpiece was measured to be 0.568 μm, which was 44% less than that of the dry workpiece, 1.016 μm. This decrease can be attributed to the lower cutting forces aided by a sharper tool edge in the cryogenic case. This also leads to reduced vibration while cutting and allows for a smoother surface to be formed. The lower surface roughness *Ra* is also due to the decreased tool wear, which was observed in the case of cryogenic machining. Less tool wear helps to maintain a sharp cutting edge for a longer time and thus yields a better surface finish of the workpiece.

## 5. Conclusions

This research studied the effect of varying the nozzle’s position on the cryogenic machining process using FEM, CFD and experimentation. This research provided a better understanding of the cryogenic effect by linking the different thermo-fluid properties of the cryogenic flow to the machining outcomes. The gaps in literature regarding the lack of optimization of the cryogenic delivery system were addressed, where the optimal nozzle position for cooling was determined using FEM, CFD and validated experimentally. The variation in nozzle distance was found to have a more significant effect than the inclination angle on the cooling of the tool–chip interface. It was shown that a smaller separation distance and inclination angle from the rake face led to an enhanced cooling effect, mainly due to the increase in velocity and turbulence near the tool–chip interface. It was also shown that a higher static pressure near the tool–chip interface led to a better cooling effect mainly due to the thin thermal boundary layer found in that region of the flow.

Experimentally, the cutting force measurements showed that the nozzle with the smallest separation distance and orientation angle led to the biggest reduction in cutting forces. The optimal case resulted in a decrease of up to 48% in the case of cryogenic machining compared to dry conditions. This was explained by the decrease in the thermal-induced softening effect which led to a decrease in tool wear and allowed the cutting tool to retain a sharp cutting edge, thus facilitating the cutting of the workpiece. Adhesion and diffusion, which highly depend on the temperature at the cutting zone, were reduced in the case of cryogenic application because of the lower cutting temperatures. Wear on the crater face due to macroscopical chipping was observed in the dry turning case, but less in the cryogenic case and this was justified by the decrease in thermal gradients and diffusion of the binder and carbon material between the adhered layer and the tool, making the insert weaker. Finally, cryogenic application led to a 44% decrease in surface roughness, due to the decrease in cutting forces and tool wear which preserved a sharp cutting edge for a prolonged period of time. Building on this work, future research can further investigate the effects of varying other nozzle parameters such as the angle from the cutting plane in order to further enhance the understanding of the cryogenic effect during machining.

## Figures and Tables

**Table 1:** Simulation cases.

Simulation	D_s_	∅
1	2 cm	15°
2	2 cm	30°
3	4 cm	15°
4	4 cm	30°

**Table 2:** Design of experiments.

	Experiment	Cryogenic Application	D_s_	∅	Duration (s)
**Orthogonal Cutting**	1	Yes	2 cm	15°	20 s
	2	Yes	2 cm	30°	
	3	Yes	4 cm	15°	
	4	Yes	4 cm	30°	
	5	No	-	-	
**Longitudinal Turning**	6	No	-	-	50 s
	7	Yes	2 cm	15°	

**Table 3:** EDS weight percentages at different spectra after orthogonal turning.

Spectrum		Weight Percentages (%wt)				
		Ti	Al	V	W	Co
1	Dry	76.9	6.4	3.9	0.6	0.1
2	Cryogenic	54.8	4.2	3.4	2.0	0.3

**Table 4:** EDS weight percentages at different spectra after longitudinal turning.

Spectrum		Weight Percentages (%wt)				
		Ti	Al	V	W	Co
3	Dry	88.1	0.4	4.9	2.3	0.1

## Data Availability

Data is contained within the article and can be requested from the corresponding author.
